# Sleep Deprivation Enhances Cocaine Conditioned Place Preference in an Orexin Receptor-Modulated Manner

**DOI:** 10.1523/ENEURO.0283-20.2020

**Published:** 2020-11-04

**Authors:** Theresa E. Bjorness, Robert W. Greene

**Affiliations:** 1 Research Service, VA North Texas Health Care System, Dallas, TX 75126-7167; 2 Department of Psychiatry, University of Texas Southwestern Medical Center, Dallas, TX 75390-9111; 3 Department of Neuroscience, Peter O’Donnell Jr. Brain Institute, University of Texas Southwestern Medical Center, Dallas, TX 75390-9111; 4 International Institute for Integrative Sleep Medicine, University of Tsukuba, Tsukuba 305-8577, Japan

**Keywords:** cocaine, conditioned place preference, mouse, orexin, sleep deprivation

## Abstract

Drug addiction and withdrawal are characterized by sleep disruption, but the effects of sleep disruption on these states are not well characterized. Sleep deprivation (SD) immediately before the cocaine conditioning trials enhanced cocaine conditioned place preference (CPP) in a dose-dependent manner (3, 8 mg/kg but not 15 mg/kg) in mice. SD immediately before the postconditioning test also enhanced cocaine CPP preference in a dose-dependent manner (8 mg/kg, but not 3, 15 mg/kg). Exposure to orexin-receptor antagonism (1 mg/kg SB 334867, an orexin 1 receptor antagonist; OX1R) just before cocaine-conditioning trials or the postconditioning test attenuated SD-enhanced preference. This suggests a potential therapeutic role for the manipulation of the orexin system to mitigate drug seeking, especially in the context of sleep loss before drug exposure.

## Significance Statement

Drugs of abuse, including cocaine, disturb sleep, and sleep disturbance has been implicated in probability of relapse; however, there have been few direct tests of sleep disturbance on drug-seeking behavior. Here, we show that acute (4 h) sleep deprivation (SD) enhances the rewarding properties of cocaine, a drug with high abuse potential. Furthermore, antagonism of orexin-system neuromodulation, involved in motivation-based arousal, reduces this SD-induced enhancement of cocaine preference.

## Introduction

Cocaine, a psychostimulant with high abuse potential because of its strong reinforcing properties (Johanson et al., 1976; Bozarth and Wise, 1985), blocks monoaminergic transporters. Of these, the dopamine transporter blockade is predominately responsible for reinforcement (Ritz et al., 1987). Cocaine is readily self-administered in non-humans to the point that unlimited access is often fatal (Johanson et al., 1976; Bozarth and Wise, 1985). These appetitive properties can be measured using conditioned place preference (CPP; Bardo and Bevins, 2000): an associative learning task in which rewarding properties of stimuli are inferred based on time spent in a context associated with a specific drug/stimuli (such as cocaine) relative to a neutral stimuli (such as saline). As expected for a drug that produces reinforcement, animals show preference for environments in which they have previously received cocaine (Mucha et al., 1982; Spyraki et al., 1982).

Acute cocaine exposure potentiates arousal by increasing sleep latency and increasing the amount of time in waking in a dose-dependent manner (Dugovic et al., 1992; Knapp et al., 2007; Bjorness and Greene, 2018a). Increases in subsequent sleep compensate for the sleep loss to the extent that there is no overall change in the amount of sleep/waking over the 24-h period in response to either acute (Bjorness and Greene, 2018a) or to several days of repeated (Dugovic et al., 1992) cocaine exposure. Non-compensated reductions in sleep are observed following withdrawal from cocaine self-administration, with decreases in non-rapid eye movement sleep emerging one week into withdrawal, decreases in rapid eye movement sleep emerging 1 d into withdrawal, and decreases in both persisting through three weeks of withdrawal (Chen et al., 2015). Thus, chronic sleep disturbance emerges after more extensive exposure to cocaine, while limited cocaine induces a sleep deprivation (SD) plus recovery response.

SD can influence drug use, as suggested by evidence that subjective sleep quality is a robust predictor of relapse to alcohol consumption (Brower et al., 2001) and that lack of Slow Wave Sleep (SWS) time recovery across abstinence is associated with relapse to cocaine use (Angarita et al., 2014). Furthermore, subjective sleep disturbance is associated with cocaine relapse following treatment in a large cohort study (Dolsen and Harvey, 2017). In rodents, chronic sleep restriction can increase motivation for cocaine (i.e., the amount of work an animal will do to obtain a cocaine reward) in a subset of animals (Puhl et al., 2013). SD also influences reward in that SD increases preference for the stimulant methylphenidate in humans (Roehrs et al., 1999) and induces preference to a low dose of amphetamine in mice (Berro et al., 2018).

The mechanism/s by which sleep loss could influence reward seeking have yet to be determined; however, the peptide neuromodulator orexin (also known as hypocretin) shows differential activity across sleep/waking states with increased activity during extended waking (i.e., SD) compared with typical waking (Estabrooke et al., 2001; Yoshida et al., 2001), is modulated by cocaine and other drugs of abuse (Thannickal et al., 2018; James et al., 2019), and influences reward seeking (Hollander et al., 2012; Gentile et al., 2018), making it an attractive candidate system. Furthermore, since orexin is heavily implicated in both maintenance of arousal (Sakurai et al., 2010) and motivated behavior (James et al., 2017), it has been hypothesized to integrate arousal and motivation (Tyree et al., 2018).

In the present study, we tested the hypothesis that acute SD enhances cocaine CPP and that the orexin system has an important role in this modulation.

## Materials and Methods

### Animals

Adult male C57BL/6 mice were obtained from Charles River Laboratories. Mice were assigned into groups (described in study 1, 2, 3, and 4 sections below) and placed into cages atop a treadmill apparatus with food and water available *ad libitum* in rooms with an ambient temperature of 22.0 ± 1°C and a 12/12 h light/dark cycle. All experiments were approved by the VA North Texas Health Care System IACUC and were in accordance with recommendations in the Guide for Care and Use of Laboratory Animals (United States National Research Council).

### Cocaine CPP

An unbiased design was used with three chambered CPP boxes (Med Associates). These boxes were unbiased in that there was no overall preference for either of the side chambers which feature different wall and flooring patterns to make them easily distinguishable. First, mice were given a preconditioning test in which they were placed into the center chamber (doors open) and allowed to explore for 20 min. Mice were excluded if they showed an innate preference for either side (as defined by >20% difference in percent time spent between sides) or if they spent more time in the center chamber than either side chamber. A subset of “excluded” mice underwent a second preconditioning test using a different CPP box (featuring different floor and wall patterns). These double pretested animals were divided evenly across groups. Next, mice underwent four conditioning trials (doors closed) in which they received cocaine (3, 8, or 15 mg/kg) or saline (vehicle control) with one, 30-min trial per day. Finally, mice were given a 20-min postconditioning test (doors open). Testing and conditioning trials occurred between zeitgeber time (ZT)4 and ZT7 and were conducted under low light level to encourage exploration. Time in each chamber was determined by IR beam break (automated) or video (manual). Based on previous reports, preference is expected for the 8 mg/kg dose (Campbell et al., 2000) and for the 15 mg/kg dose (Nomikos and Spyraki, 1988) but not for the 3 mg/kg dose (Zachariou et al., 2001). While conditioning protocols vary, two trials are expected to be sufficient to support the development of cocaine preference (McClung et al., 2005; Graham et al., 2009).

### Cocaine CPP study 1 (Fig. 1*A*)

For each cocaine dose (3, 8, 15 mg/kg), two groups of animals were compared [group names are designated based on the sleep parameters (SD or undisturbed; noSD) before each set of conditioning trials (cocaine, Coc; or saline, Sal)]. Both groups received cocaine and saline on alternating days. The experimental group of mice underwent SD for 4 h immediately before cocaine conditioning trials and, on alternate days, were undisturbed before saline conditioning trials (SD Coc, noSD Sal). The control group was sleep deprived for 4 h immediately before the saline conditioning trials (noSD Coc, SD. Sal) but was undisturbed before the cocaine conditioning trials. An additional experiment was used to test whether SD is sufficient to induce preference. Subjects received saline on both side chambers with (SD Sal, noSD Sal) or without SD (noSD Sal, noSD Sal) before saline-conditioning trials on one side of the box. Animals were weighed before each conditioning trial. There was no difference in preconditioning relative time values (side A – side B) between groups for any of the doses (0 mg/kg, *p* = 0.96; 3 mg/kg, *p* = 0.98; 8 mg/kg, *p* = 0.99; 15 mg/kg, *p *= 0.71).

### Cocaine CPP study 2 (Fig. 2*A*)

For each cocaine dose (3, 8, 15 mg/kg), two groups of animals were compared [group names are designated based on the sleep parameters, SD undisturbed (noSD), before the postconditioning test]. Both groups received cocaine and saline on alternating days. In this study, the experimental group of mice was sleep deprived on only one occasion, i.e., 4 h immediately before the post-test (SD), while a control group was undisturbed (noSD). There was no difference in preconditioning relative time values between groups for any of the doses (3 mg/kg, *p *= 0.99; 8 mg/kg, *p = *0.73; 15 mg/kg, *p* = 0.68).

### Orexin-receptor antagonism during conditioning and SD-enhanced cocaine CPP study 3 (Fig. 3*A*)

Subsets of mice receiving 3 or 8 mg/kg cocaine were injected with the orexin 1 receptor (OX1R) antagonist SB 334867 (1 mg/kg; SB) or vehicle (Veh) 15 min before the conditioning trials (group names are designated based on sleep parameters and OX1R-antagonism status before each conditioning test). The experimental group was sleep deprived for 4 h and given SB before cocaine conditioning trials (SD SB Coc, noSD Veh Sal), while a control subset of mice was sleep deprived and given SB before the saline paired trials (noSD Veh Coc, SD SB Sal). For the 8 mg/kg dose, a third subset of mice was injected with SB before the cocaine paired trials but was not sleep deprived, serving as an OX1R antagonist-only control (noSD SB Coc, noSD Veh Sal). There was no difference in preconditioning relative time values between groups at either dose (3 mg/kg, *p *= 0.9; 8 mg/kg, *p* = 0.99). SB 334867 was chosen on the basis of its common use in addiction-related studies, while the cocaine doses chosen were based on the doses in which there were significant group differences in study 1 (3, 8 mg/kg).

### OX1R antagonism after conditioning and SD-enhanced cocaine CPP study 4 (Fig. 4*A*)

Subsets of mice receiving 8 mg/kg cocaine were injected with SB 15 min before the post-test. The experimental group was sleep deprived for 4 h before receiving SB (SD SB), while the control group was undisturbed before receiving SB (noSD SB). There was no difference in preconditioning relative time values between groups (*p *= 0.98). The cocaine dose chosen was based on the dose in which there was a significant group difference in study 2 (8 mg/kg).

### SD

Mice were sleep deprived using the treadmill method (Bjorness et al., 2009) in which waking is enforced through slow walking; the belt speed was ∼3 cm/s (for comparison belt speeds of ∼20 cm/s are used for exercise; Um et al., 2011). SD began early in the light phase (ZT0–ZT2) and concluded immediately before CPP conditioning or testing for a total of 4 h. Food and water were available throughout the SD period. Four hours of SD was used since this duration reliably induces a homeostatic response as measured by an increase in slow wave activity (SWA; 0.5–4.5 Hz) during slow wave sleep (Bjorness and Greene, 2018b). Furthermore, this duration does not increase expression of glucocorticoid-related genes as determined by transcriptome analysis of cortical tissue (Bjorness et al., 2020).

### Drugs

Cocaine hydrochloride (Sigma-Aldrich) was dissolved in sterile saline and injected in doses of 3, 8, or 15 mg/kg with a volume of ≤0.1 ml. Sterile saline was used as the vehicle control. SB 334867 (Sigma-Aldrich) was dissolved in dimethyl sulfoxide (DMSO), then diluted in sterile water (10% DMSO). DMSO diluted with sterile water was used as the vehicle control.

### Outcome measures

**Figure 1.**
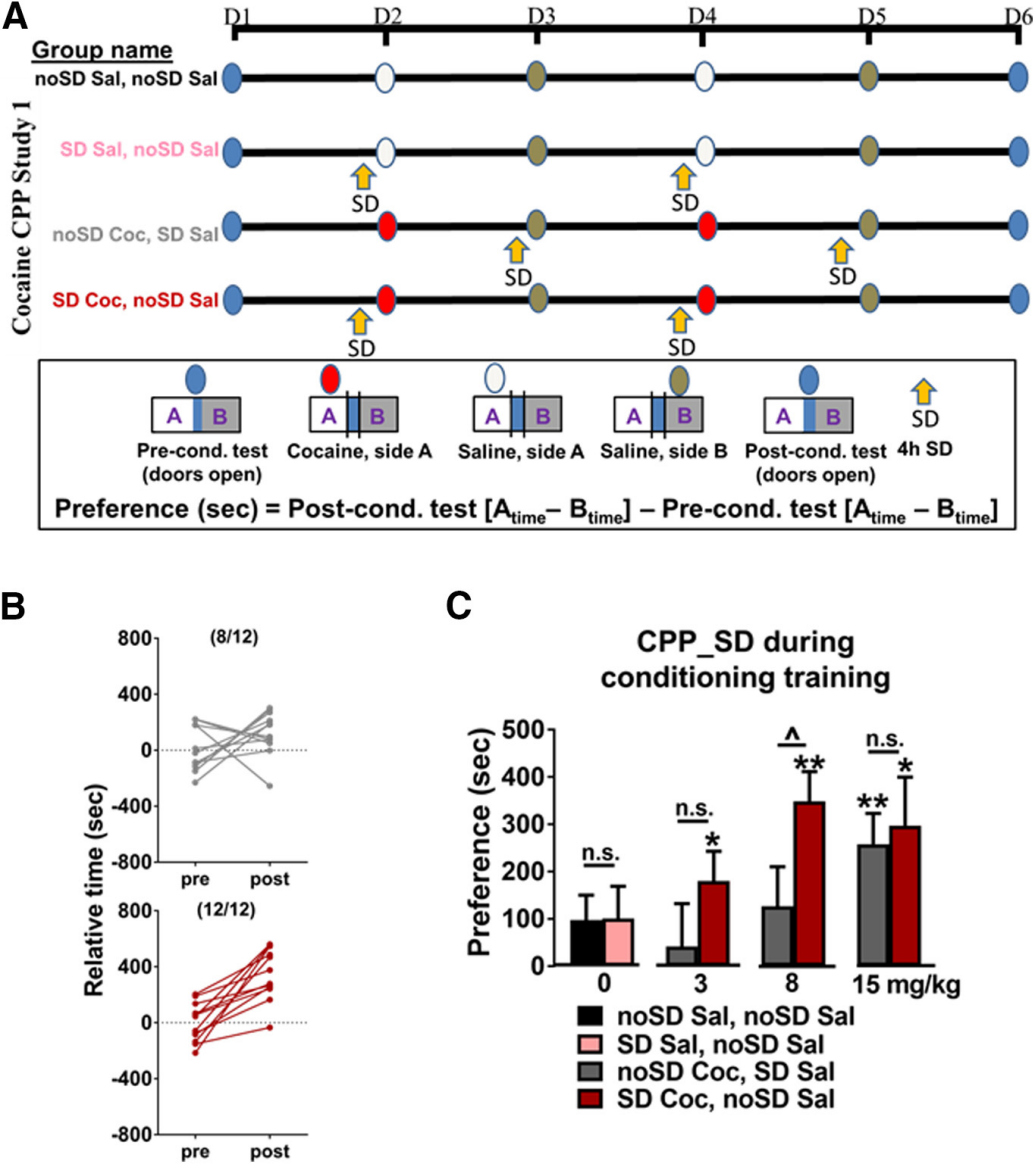
***A***, Experimental timeline of cocaine CPP study 1. ***B***, Following conditioning to 8 mg/kg cocaine, most animals undisturbed before cocaine conditioning trials spent more time in the cocaine-paired side following conditioning as expected as compared with their preconditioning test times (top), which shifted to all animals when SD occurred immediately before cocaine conditioning trials (bottom). ***C***, SD immediately before cocaine conditioning trials induced preference to 3 mg/kg cocaine and enhanced preference to 8 mg/kg cocaine without altering preference to 15 mg/kg cocaine. SD in the absence of cocaine (0 mg/kg) did not induce preference. Asterisks above columns indicate preference (as determined by a significant difference from 0), the carrot between columns indicates a significant difference between groups, and n.s. indicates a lack of significant difference between groups. Bars indicate group average, error bars indicate standard error of the mean (SEM).

**Figure 2.**
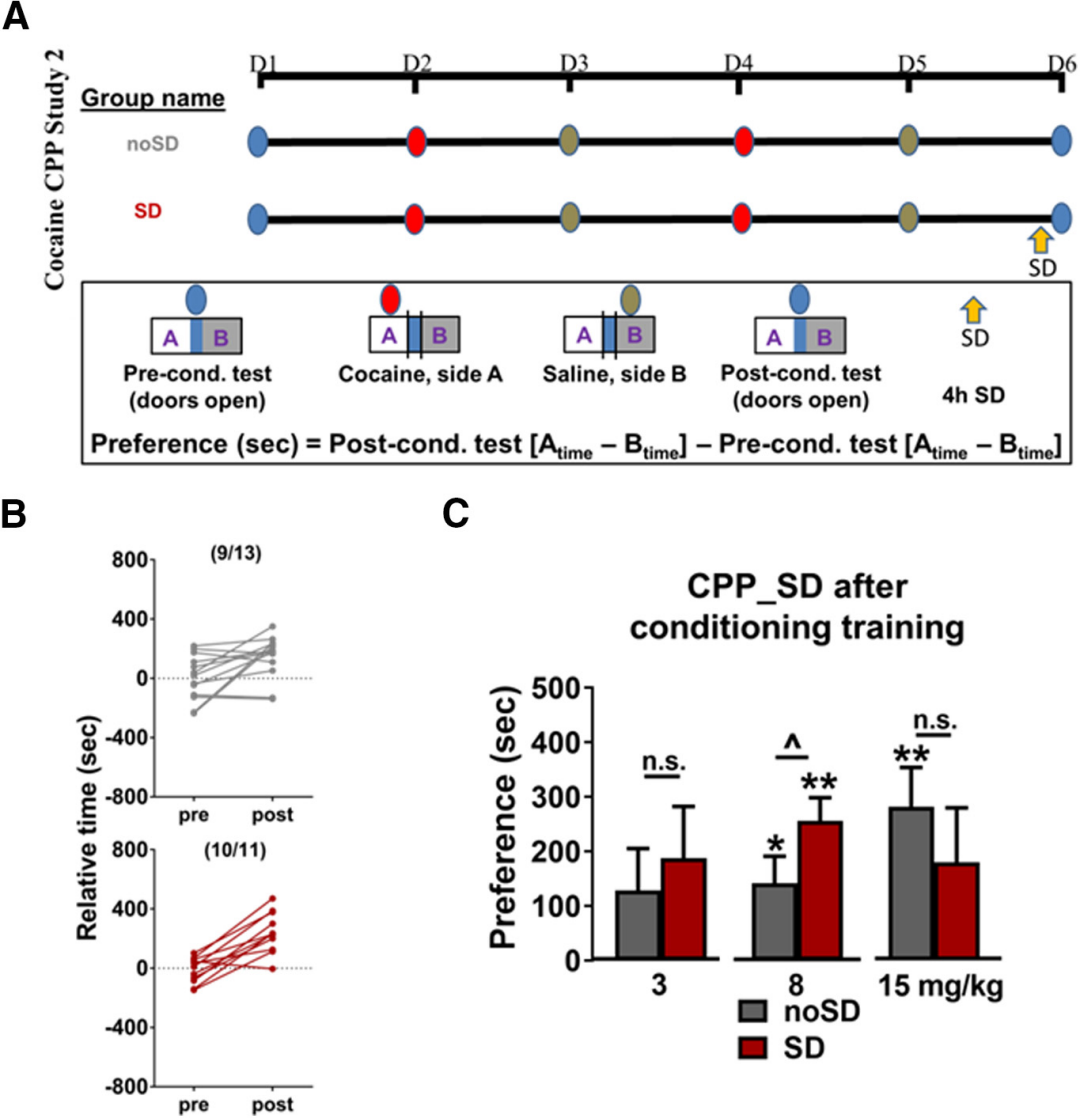
***A***, Experimental timeline for the cocaine CPP study 2. ***B***, Following conditioning to 8 mg/kg cocaine, most animals undisturbed before the postconditioning test spent more time in the cocaine-paired side as compared with their preconditioning test times (top), which shifted to a higher proportion of animals when SD occurred immediately before the postconditioning test (bottom). ***C***, SD immediately before postconditioning test induced a non-significant trend toward preference to 3 mg/kg cocaine and enhanced preference to 8 mg/kg cocaine, while reducing preference to a non-significant trend to 15 mg/kg cocaine. Asterisks above columns indicate preference (as determined by a significant difference from 0), the carrot between columns indicates a significant difference between groups, and n.s. indicates a lack of significant difference between groups. Bars indicate group average, error bars indicate SEM.

**Figure 3.**
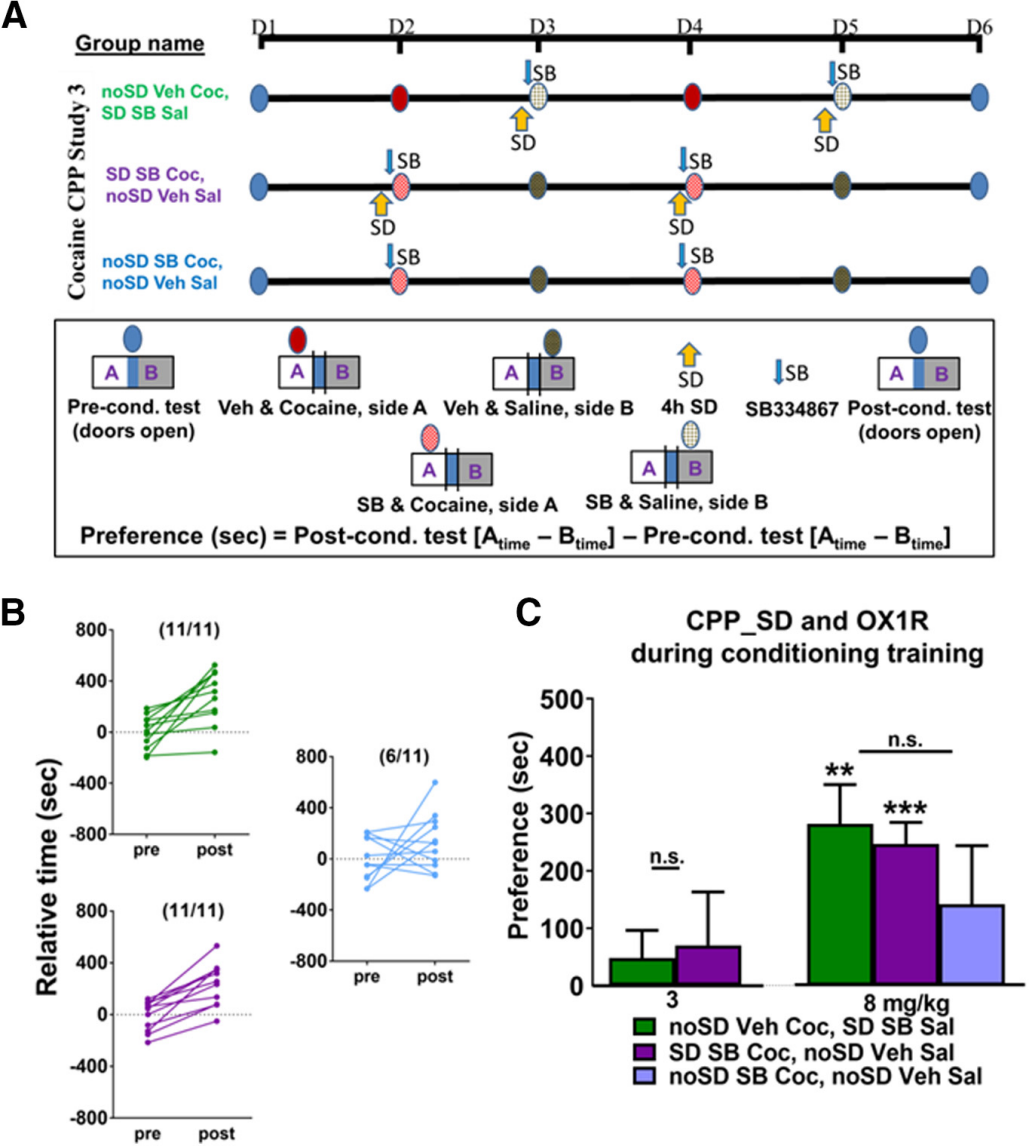
***A***, Experimental timeline for the cocaine CPP study 3. ***B***, All animals spent more time in the cocaine-paired side following conditioning as compared with their preconditioning test values when Veh was administered before the cocaine trials (top) or when SB 334867 was administered immediately following SD (bottom); however, only a subset of animals spent more time in the cocaine-paired side when SB 334867 was administered in the absence of SD (right side). ***C***, OX1R antagonism before cocaine conditioning trials blocked the SD-induced preference to 3 mg/kg cocaine and the SD-induced enhanced preference to 8 mg/kg cocaine, while OX1R antagonism in the absence of SD prevented the acquisition of preference to 8 mg/kg cocaine. Asterisks above columns indicate preference (as determined by a significant difference from 0), and n.s. indicates a lack of significant difference between groups. Bars indicate group average, error bars indicate SEM.

**Figure 4.**
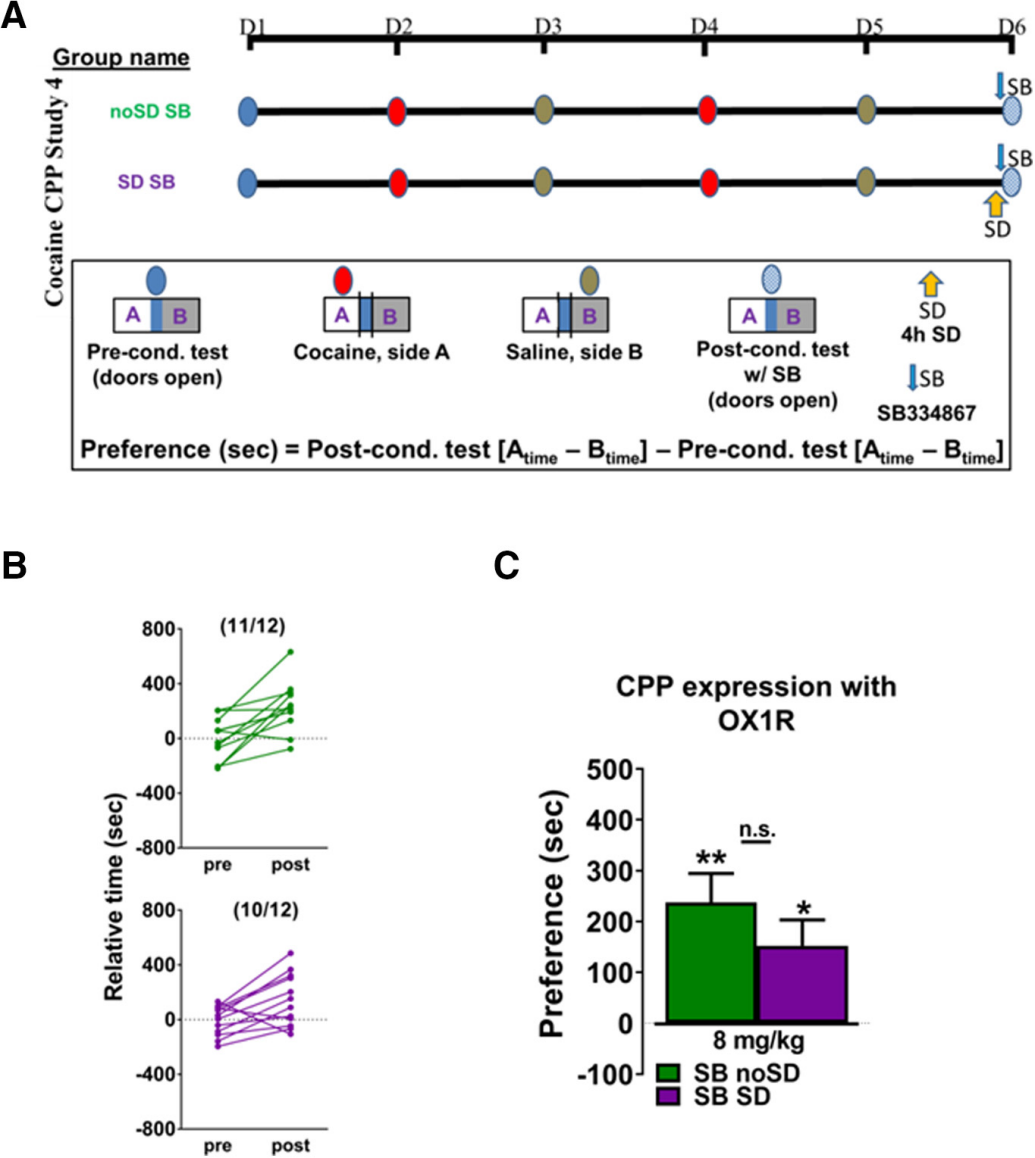
***A***, Experimental timeline for cocaine CPP study 4. ***B***, Most animals spent more time in the cocaine-paired side following conditioning as compared with their preconditioning test values both when undisturbed animals were administered SB 334687 before the postconditioning test (top) and when sleep-deprived animals were administered SB 334867 before the postconditioning test (bottom). ***C***, OX1R antagonism prevents the SD-induced increase in preference to 8 mg/kg cocaine, although both groups show preference for the cocaine-paired side. Asterisks above columns indicate preference (as determined by a significant difference from 0), and n.s. indicates a lack of significant difference between groups. Bars indicate group average, error bars indicate SEM.

The main outcome measure for cocaine CPP was preference score which was calculated as preference (s) = postconditioning test (side A_time_ – side B_time_) – preconditioning test (side A_time_ – side B_time_), with A side conditioning in trials 1, 3 and B side in trials 2, 4. For studies 1 and 2, control and experimental groups were compared using one tailed unpaired *t* tests (3, 8, or 15 mg/kg cocaine) or two tailed unpaired *t* test (0 mg/kg cocaine). For studies 3 and 4, control and experimental groups were compared using two tailed unpaired *t* test (3 mg/kg study 3, 8 mg/kg study 4) or one-way ANOVA with Sidak correction for multiple comparisons (8 mg/kg study 4). One tailed *t* tests were used for comparisons in which there is literature support for an effect of SD on preference outcomes, while two tailed *t* tests were used for comparisons lacking direct literature support for an effect of SD on preference outcomes. Additionally, preconditioning relative time values (time in side to be paired with cocaine – time in side to be paired with saline) were also compared for each experiment to ensure equal balancing (with respect to preconditioning preference time) of groups before cocaine exposure. For comparison to a theoretical mean of 0, a two-tailed one sample *t* test was used; positive values significantly different from 0 indicate preference. All statistical analyses were performed using GraphPad prism. Values are given as average ± SEM, and significance is set at *p < *0.05. For all studies, preconditioning and postconditioning relative values are shown (Figs. 1*B*, 2*B*, 3*B*, 4*B*) for the 8 mg/kg dose to illustrate variability between animals within each group alongside the general pattern of increased time in the cocaine-paired side.

## Results

### Cocaine CPP, study 1

To examine the effects of SD on cocaine CPP, we compared CPP in experimental and control groups of mice. Both groups were alternately (every other day) conditioned to cocaine and saline (Coc, Sal); however, the experimental group’s cocaine conditioning was preceded by 4 h of SD (SD. Coc, noSD Sal) whereas the control group’s saline conditioning was preceded by SD (noSD Coc, SD. Sal) as illustrated in Figure 1*A*. To control for the potential effects of SD in the absence of cocaine conditioning, an additional two cohorts of mice received saline every day either with (SD. Sal, noSD Sal) or without SD (noSD Sal, noSD Sal).

As expected, most animals (8/12 noSD Coc, SD. Sal, 12/12 SD. Coc, noSD Sal) that received the 8 mg/kg dose of cocaine showed an increase in the time spent in the cocaine-paired side, tested after conditioning (Fig. 1*B*); however, SD immediately before cocaine conditioning trials resulted in an increase in time spent on the cocaine-paired side over animals experiencing SD preceding saline (Fig. 1*C*; Table 1). In the absence of SD, animals did not show any preference for the 3 mg/kg cocaine-conditioned side. In contrast, SD immediately before cocaine conditioning trials induced preference to a 3 mg/kg dose of cocaine (Fig. 1*C*; Table 1). SD did not influence preference to a 15 mg/kg dose of cocaine, a possible ceiling-like effect (Fig. 1*C*; Table 1). Controls (noSD Coc, SD Sal) showed preference at 15 mg/kg but not at 8 mg/kg, possibly because of high variability driven by one animal (not identified as an outlier when using the ROUT method; GraphPad Prism). SD in the absence of cocaine did not induce preference (0 mg/kg; Fig. 1*C*; Table 1).

**Table 1:** Cocaine CPP, study 1

	0 mg/kg		3 mg/kg	
Study 1	noSD Sal, no SD. Sal	SD. Sal, noSD Sal	noSD Coc, SD. Sal	SD. Coc, noSD Sal
*N*	12	12	10	11
Mean ± SEM	97.81 ± 52.49	100.6 ± 63.38	41.83 ± 90.53	180.4 ± 62.75
95% CI	−17.73−213.4	−49.92−251.1	−163−246.6	40.56−320.2
*p* value	0.09	0.17	0.655	0.0165
Stat used for comparison	One sample *t* test	One sample *t* test	One sample *t* test	One sample *t* test
	8 mg/kg		15 mg/kg	
	noSD Coc, SD. Sal	SD. Coc, noSD Sal	noSD Coc, SD. Sal	SD. Coc, noSD Sal
*N*	12	12	12	12
Mean ± SEM	126.4 ± 83.62	348.7 ± 62.95	257.9 ± 65.4	284.9 ± 94.67
95% CI	−57.64−310.5	210.1−487.2	114−401.9	76.5−493.2
*p* value	0.1588	0.0002	0.0023	0.0119
Stat used for comparison	One sample *t* test	One sample *t* test	One sample *t* test	One sample *t* test
Group comparison	0 mg/kg	3 mg/kg	8 mg/kg	15 mg/kg
Difference between means	−2.521 ± 49.26	−138.5 ± 108.5	−222.3 ± 104.7	−26.93 ± 115.1
95% CI	−104.7−99.63	−365.6−88.46	−439.3 to −5.173	−265.6−211.7
*p* value	0.9596	0.11	0.0226	0.4085
Stat used for comparison	Unpaired *t* test, two tailed	Unpaired *t* test, one tailed	Unpaired *t* test, one tailed	Unpaired *t* test, one tailed

### Cocaine CPP, study 2

To examine the effects of SD on cocaine CPP after conditioning has been established, cocaine-conditioned animals underwent SD for 4 h immediately before the postconditioning test. The control group was similarly conditioned but remained undisturbed before testing for CPP (Fig. 2*A*).

As expected, most animals (*n* = 9/13 noSD; 10/11 SD) that received the 8 mg/kg dose of cocaine showed an increase in the time spent in the cocaine-paired side from the preconditioning to postconditioning tests (Fig. 2*B*). SD immediately before the postconditioning test induced a non-significant trend toward preference to a 3 mg/kg dose of cocaine (Fig. 2*C*; Table 2) and significantly increased preference to an 8 mg/kg dose of cocaine (Fig. 2*C*; Table 2). There was no difference in preference between groups to a 15 mg/kg dose of cocaine (Fig. 2*C*; Table 2), but the sleep-deprived group showed a non-significant trend toward preference for the cocaine-paired side.

**Table 2:** Cocaine CPP, study 2

	3 mg/kg		8 mg/kg		15 mg/kg	
Study 2	noSD	SD	noSD	SD	noSD	SD
*N*	12	11	13	11	12	12
Mean ± SEM	128.3 ± 76.91	187.4 ± 94.94	141.3 ± 49.55	256 ± 42.39	281.8 ± 72.06	179.6 ± 100.1
95% CI	−41.02−297.5	−24.19−398.9	33.35−249.3	161.6−350.5	123.2−440.4	−40.84−400
*p* value	0.1236	0.0767	0.0146	0.0001	0.0024	0.1005
Stat used for comparison	One sample *t* test	One sample *t* test	One sample *t* test	One sample *t* test	One sample *t* test	One sample *t* test

Group comparison	3 mg/kg		8 mg/kg		15 mg/kg	
Difference between means	−59.11 ± 121.3		114.7 ± 66.55		102.3 ± 123.4	
95% CI	−311.3−193.1		−23.31−252.7		−153.6−358.1	
*p* value	0.3155		0.0494		0.2081	
Stat used for comparison	Unpaired *t* test, one tailed		Unpaired *t* test, one tailed		Unpaired *t* test, one tailed	

### OX1R antagonism during conditioning and SD-enhanced cocaine CPP, study 3

A previous observation indicates that SB 334867 during conditioning attenuates cocaine CPP (Rao et al., 2013). To examine the effects of OX1R antagonism on SD-induced enhancement of cocaine CPP, we compared CPP in an experimental and control group as in study 1, except that immediately following SD, but before each training session, animals received the OX1R antagonist SB 334867 (SB) or vehicle (Veh) on alternating days (Fig. 3*A*). To test for OX1R antagonism effects in the absence of SD, SB 334867 was also given to cohort that did not undergo SD.

In response to 8 mg/kg cocaine, all sleep-deprived animals showed an increase in the time spent on the cocaine-paired side; however, only a subset (6/11) of animals receiving OX1R antagonism in the absence of SD showed this relative increase (Fig. 3*B*). In contrast to the observations of study 1 in which SD induced cocaine CPP to a 3 mg/kg dose of cocaine, SB 334867 prevented this induction. Finally, SB 334867 prevented preference in the absence of SD and blocked SD-induced enhancement of preference to a 8 mg/kg dose of cocaine as determined by a lack of difference between groups; however SD animals treated with SB 334867 did show preference for the cocaine-paired side suggesting that SD-dependent enhancement is reduced but not entirely prevented (Fig. 3*C*; Table 3).

**Table 3:** Cocaine CPP, study 3

	3 mg/kg		8 mg/kg		
Study 3	noSD Veh Coc, SD SB Sal	SD SB Coc, noSD Veh Sal	noSD Veh Coc, SD SB Sal	SD SB Coc, noSD Veh Sal	noSD SB Coc, noSD Veh Sal
*N*	11	12	11	11	11
Mean ± SEM	48.12 ± 48.24	70.25 ± 93.15	282 ± 68.44	246.8 ± 37.69	141.8 ± 102.1
95% CI	−59.36−155.6	−134.8−275.3	129.5−434.5	162.8−330.7	−85.82−369.4
*p* value	0.342	0.4666	0.0021	<0.0001	0.1953
Stat used for comparison	One sample *t* test	One sample *t* test	One sample *t* test	One sample *t* test	One sample *t* test

Group comparison	3 mg/kg noSD Veh Coc, SD SB Sal vs SD SB Coc, noSD Veh Sal	8 mg/kg noSD Veh Coc, SD SB Sal vs SD SB Coc, noSD Veh Sal	8 mg/kg noSD Veh Coc, SD SB Sal vs noSD SB Coc, noSD Veh Sal		
Difference between means	22.13 ± 107.8	35.25 ± 105	140.2 ± 105		
95% CI	−202.1−246.4	−211.9−282.4	−106.9−387.4		
*p* value	0.8394	0.9321	0.3467		
Stat used for comparison	Unpaired *t* test, two tailed	One-way ANOVA with Sidak correction for multiple comparisons	One-way ANOVA with Sidak correction for multiple comparisons		

### OX1R antagonism after conditioning and SD-enhanced cocaine CPP, study 4

Previous observations indicate that SB after conditioning does not influence cocaine CPP (Sharf et al., 2010; Sartor and Aston-Jones, 2012). The effect of OX1R antagonism together with SD on cocaine CPP after establishment of conditioning was examined by comparing CPP in an experimental and control group of mice as in study 2, except that all animals received the OX1R antagonist before the postconditioning test (Fig. 4*A*).

A similar majority of undisturbed or sleep-deprived animals administered SB 334867 just before the postconditioning test, showed an increase in the time spent on the cocaine-paired side (Fig. 4*B*). The antagonism of OX1R after establishment of conditioning was sufficient to prevent the SD-induced enhancement of cocaine CPP to an 8 mg/kg dose of cocaine (Fig. 4*C*; Table 4).

**Table 4:** Cocaine CPP, study 4

Study 4	8 mg/kg	
	noSD SB	SD SB
*N*	12	12
Mean ± SEM	237.4 ± 57.03	152.2 ± 50.88
95% CI	111.8−362.9	40.17−264.1
*p* value	0.0016	0.0123
Stat used for comparison	One sample *t* test	One sample *t* test

Group comparison	8 mg/kg	
Difference between means	−85.21 ± 76.42	
95% CI	−243.7−73.28	
*p* value	0.2769	
Stat used for comparison	Unpaired *t* test, two tailed	

### SD shifts the cocaine CPP dose-response curve leftward in an orexin-influenced manner

**Figure 5.**
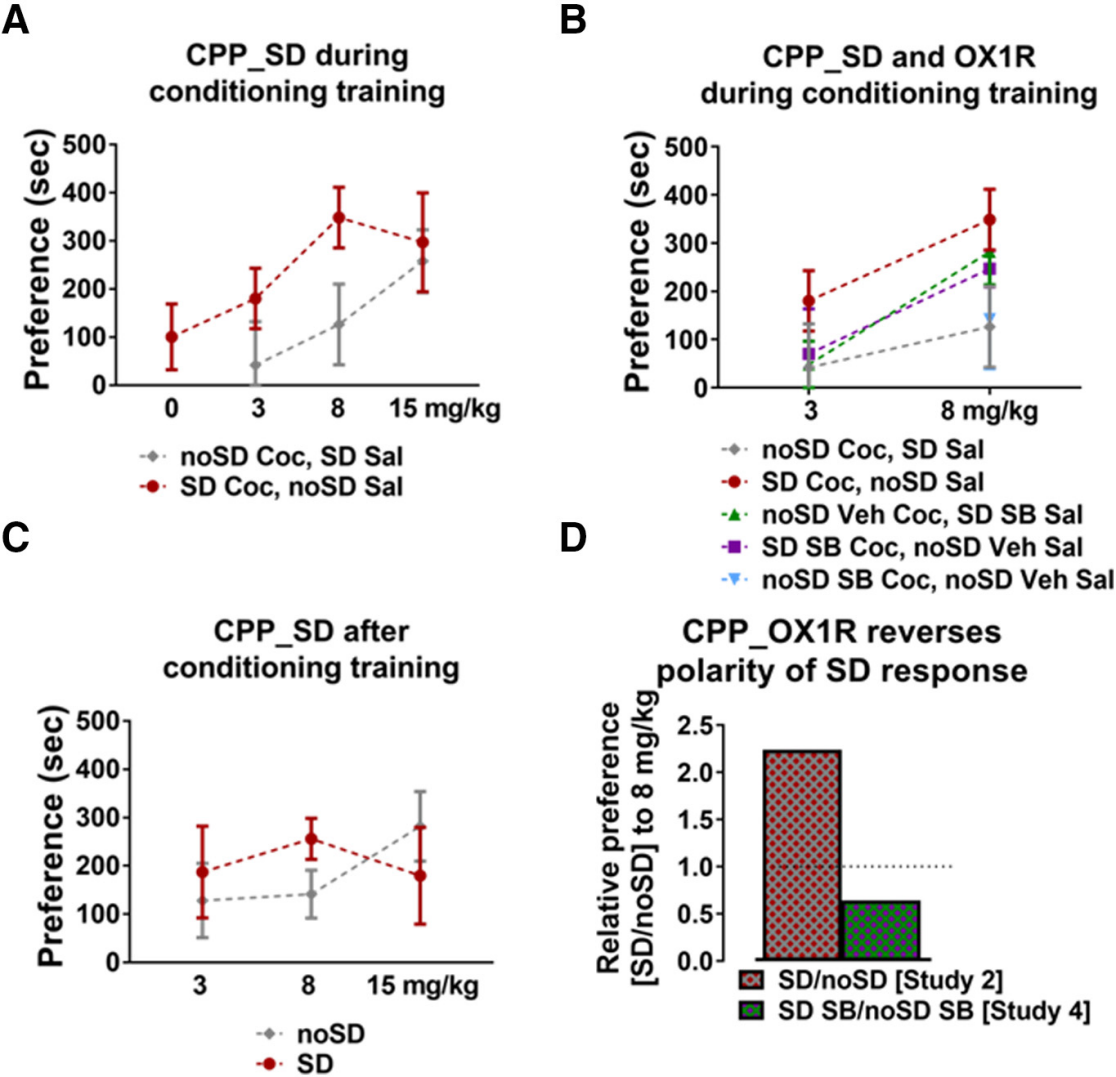
***A***, Dose-response plot of preference from cocaine CPP study 1 in which SD shifts the curve leftward (data replotted from Fig. 1*C*). ***B***, OX1R antagonism mitigates the SD-induced shift in the dose-response curve (data replotted from Figs. 1*C*, 3*C*). ***C***, Dose-response plot of preference from cocaine CPP study 2 in which SD shifts the curve leftward, although to a lesser degree than under cocaine CPP study 1 (data replotted from Fig. 2*C*). ***D***, The SD enhancement of relative preference, as determined by the ratio of average preference in SD and noSD groups (from study 2) and indicated by >1 value (left bar), is blocked by OX1R antagonism (SD SB/noSD SB from study 4; right bar). Notably, the relative preference of <1 under OX1R antagonism indicates that OX1R antagonism in the presence of SD reduces relative preference, while OX1R antagonism in the absence of SD increases relative preference.

SD immediately before cocaine-conditioning trials shifts the preference dose-response curve leftward (Fig. 5*A*) which is consistent with an increasing sensitivity to the rewarding properties of cocaine. However, OX1R antagonism immediately before the cocaine-conditioning trials reduces this shift (Fig. 5*B*). An SD-related leftward shift in the dose-response curve is also apparent when SD occurs immediately before the postconditioning test (Fig. 5*C*) and it is reduced by OX1R antagonism. Unexpectedly, OX1R antagonism in undisturbed animals before the postconditioning test (study 4; noSD SB) led to preference values similar to that of sleep-deprived animals in the absence of OX1R antagonism (study 2; SD), thereby reversing the polarity of the effect of SD as determined by dividing the group average preference score of the sleep-deprived group by the group average preference score of the undisturbed group [i.e., (study 2, SD/noSD); (study 4, SD SB/noSD SB)]. A score above 1 indicates that SD results in a higher relative preference score compared with the undisturbed condition, while a score below 1 indicates that SD results in a lower relative preference score compared with the undisturbed condition. Statistical comparisons across studies were not performed because of data collection constraints (see limitations paragraph within Discussion) so these comparisons are observational in nature and should be interpreted with caution.

## Discussion

SD enhanced cocaine CPP in a dose-dependent manner resulting in a leftward shift in the dose-response curve, indicating SD increased the rewarding properties of cocaine. This shift was more pronounced when SD occurred immediately before cocaine exposure compared with SD after cocaine conditioning was already established, consistent with a greater SD-induced enhancement of acquisition of preference than of its expression. OX1R antagonism reduced the SD-induced enhancement of both acquisition and expression.

On the low end of the dose-response curve, SD induced preference to a subthreshold dose of cocaine, which is similar to a previously reported SD-dependent induction of preference to subthreshold amphetamine (Berro et al., 2018), suggesting SD may increase sensitivity to psychostimulants in general. On the high end of the dose-response curve, SD did not alter preference to a sensitizing dose of cocaine, possibly because of a ceiling effect and/or, an aversive effect elicited by higher doses of cocaine.

Most groups showed preference for the 8 mg/kg dose of cocaine, a dose in which preference is expected (Campbell et al., 2000); however, animals sleep deprived before saline conditioning trials (noSD Coc, SD Sal, study 1) did not reach statistical significance for preference despite the majority of animals showing an increase in time spent on the cocaine-paired side from the pre to postconditioning tests (8/12). This lack of preference is likely attributable to high variability in preference scores relative to the group average (126.4 ± 83.6) and is driven by a single animal as can be seen in Figure 1*B*, although this animal does not qualify as an outlier. As can be seen from the raw data plots with the 8 mg/kg dose across studies, most animals show an increase in relative time in the cocaine-paired side from pre to postconditioning, although not all animals do so. We cannot explain the source of the individual differences, but these are consistent with individual differences seen with locomotor sensitization to cocaine (Hooks et al., 1991; Allen et al., 2007) and cocaine self-administration (Glick et al., 1994; Griffin et al., 2007).

The ability of OX1R antagonism to reduce SD-induced enhancement of cocaine CPP is consistent with the well-known role of orexin in motivated behavior (James et al., 2017) and maintenance of arousal (Sakurai et al., 2010). Orexin neuronal activity increases during SD (Estabrooke et al., 2001) as does orexin release (Yoshida et al., 2001). Furthermore, orexin agonists promote cocaine self-administration (España et al., 2011), while antagonism of orexin activity can reduce reward behavior (Sartor and Aston-Jones, 2012; Rao et al., 2013; Shaw et al., 2017).

The SD-induced enhancement of cocaine CPP is consistent with previous studies in which SD increases preference of methylphenidate in humans (Roehrs et al., 1999) and induces preference to a low dose of amphetamine in rodents (Berro et al., 2018). However, there are several additional studies that would be of interest in further delineating the ability of sleep loss to influence reward behavior. First, thus far, all studies have used stimulants so the generalizability of the SD-induced enhancement of preference across drug class is unknown. Additionally, the time course of this enhancement preference is unclear. A long-term enhancement of preference would likely be more relevant to the development of addiction than if the SD-induced enhancement is quickly lost. Finally, it is unknown whether SD-induced enhancement of stimulant reward is sustained in drug experienced animals since existing studies have included drug naive rodents or non-dependent humans.

These studies had several limitations. First, study 4 lacked a vehicle control group; a control group of noSD SB was used for the experimental group SD SB in which the ability of OX1R-antagonism to counter SD-induced enhancement of cocaine CPP to an 8 mg/kg dose of cocaine (from study 2) was tested. DMSO was diluted to reduce the concentration below that which behavioral effects are observed (Cavas et al., 2005). However, the effect of vehicle alone on CPP expression was not determined so the possibility that SD-induced enhancement of cocaine CPP was reduced by the vehicle cannot be excluded. Another limitation relates to the lack of direct statistical comparisons across related studies (studies 1 and 3, studies 2 and 4) because of the manner of data collection. Within each study, control and experimental animals were littermates, and data were collected concurrently across groups with multiple sets of control and experimental animals collected for each study; however, there was a considerable time lag between data collection of related studies. A superior design would have included concurrent data collection for related studies so that these could be directly compared. Additionally, since activity measures are not available for conditionings performed with all of the CPP boxes, we cannot exclude a possibility that enhanced preference is associated with an increase in locomotor activity; however, we have previously shown that acute SD does not influence the magnitude of locomotor sensitization to cocaine (Bjorness and Greene, 2018b) so an SD-dependent increase in locomotor activity is not expected. Finally, the current experiments did not include female subjects so it is unknown whether gender influences SD enhancement of cocaine CPP.

In conclusion, acute SD increases the rewarding properties of cocaine in a cocaine dose-dependent manner as measured by the CPP task which suggests that sleep loss may facilitate the transition toward addiction. OX1R antagonism reduces this effect, suggesting a potential therapeutic avenue for careful consideration as an aid in abstinence maintenance. Recently, Suchting and colleagues provided preliminary proof-of-concept for use of orexin receptor antagonism in individuals with cocaine use disorder (Suchting et al., 2020). Although the study design precluded an assessment of the efficacy of a OX1R/OX2R antagonist, there is evidence for its having improved objective sleep (actigraphy) and self-reported craving measures (Cocaine Craving Questionnaire), suggesting the clinical relevance for our findings.

## Synthesis

Reviewing Editor: Leandro Vendruscolo, The Scripps Research Institute Decisions are customarily a result of the Reviewing Editor and the peer reviewers coming together and discussing their recommendations until a consensus is reached. When revisions are invited, a fact-based synthesis statement explaining their decision and outlining what is needed to prepare a revision will be listed below. The following reviewer(s) agreed to reveal their identity: Brooke Schmeichel, Yanhua Huang.

The reviewers were positive about the study, but they made several pertinent comments that can be seen in their review below. Additional experiments are not required; all of the reviewers’ critiques can be addressed with text changes.

## Author Response

The reviewers were positive about the study, but they made several pertinent comments that can be seen in their review below. Additional experiments are not required; all of the reviewers’ critiques can be addressed with text changes.

We thank the reviewers for their thoughtful critiques and helpful suggestions.

Reviewer 1

The authors set out to test whether sleep-deprivation had an effect on cocaine-induced CPP, and if so, the degree to which hypocretin/orexin modulation participates in sleep-deprived enhancement of cocaine-CPP. The question of the effects of sleep deprivation on cocaine consumption is an interesting one, and as the authors point out, has ramifications on treatment plans for stimulant substance use disorders. The hypotheses posed are likely of high interest to the addiction research field. Although a considerable amount a work went into the execution of these studies, they appear not entirely well-planned or designed, tempering this reviewer’s enthusiasm for the results. In particular, some of the study design choices (e.g., length of sleep-deprivation, length of conditioning for CPP, dose of hypocretin-antagonist tested), are not adequately justified or thought out. Similarly, the analyses of the data and interpretation are simple, yet confused. The authors need to re-work this manuscript with a richer analyses and interpretation of the data. Please consider the following comments:

1. The title does not correctly state their results given they did not manipulate the “dose” of sleep-deprivation, but rather they tested effects of sleep-deprivation on dose-dependent cocaine-induced CPP.

We modified the title to reflect the dose-dependence of cocaine not sleep deprivation duration. The new title is ‘Sleep deprivation enhances conditioned place preference in a cocaine dose-dependent manner’.

2. All text referring to “orexin antagonism” should be changed to “orexin-receptor antagonism.”

We apologize for the sloppy wording. We now use orexin-receptor (OX1R) antagonism.

3. The authors should be careful about relating cocaine-withdrawal-associated sleep literature to that of acute-cocaine-associated sleep literature (particularly in the introduction) and how it relates to their sleep-deprivation studies using acute cocaine exposure.

We agree and have added the sentence ‘Thus, chronic sleep disturbance emerges after more extensive exposure to cocaine, while limited cocaine induces a sleep deprivation plus recovery response.’

4. Limited cocaine exposure (2 of 4 days) in the establishment of CPP used in these studies is a weakness of the study. The authors may want to justify such a short phase of place conditioning.

There is no effect of the number of conditioning trials (1-4) on effect size for cocaine CPP (Bardo et al., 1995) and a 2 trial CPP is commonly used (for example, McClung et al., 2005; Graham et al., 2009; Schindler et al., 2012; Lui et al., 2016). We have added the statement ‘While conditioning protocols vary, two trials are expected to be sufficient to support the development of cocaine preference (McClung et al., 2005; Graham et al., 2009)’ as justification for the use of this design within the Cocaine Conditioned Place Preference section of the methods. Furthermore, since we expected sleep deprivation to enhance cocaine CPP such that sleep deprived animals would be more sensitive to the rewarding properties of cocaine (as in the case of the subthreshold dose of 3 mg/kg) lower cocaine exposure (in the case of the number of conditioning trials) would be a better test of this hypothesis. An expert in rodent behavior was consulted during the design of this project and is listed in the acknowledgements statement.

4. (continued). It appears in Figure 2 that they achieved cocaine-induced CPP only at the high dose of 15mg/kg. This is concerning for the interpretation of the sleep-deprivation data.

Preference was achieved by both groups at the 8 mg/kg dose as indicated by the asterisks.

5. CPP testing/pairing was conducted during light phase. Participation of hypocretin-related modulation of CPP may be affected by light/dark cycle. Could your hypocretin manipulations be compromised because of light-phase testing times (ZT4-7)?

As the reviewer points out, orexin (hypocretin) is modulated by behavioral state which itself is modulated by circadian phase with orexin levels higher during the dark (active) phase compared to the light (inactive) phase. However, orexin levels and orexin neuronal activity increase with sleep deprivation (Estabrooke et al., 2001; Yoshida et al., 2001) such that orexin would be expected to be higher in sleep deprived animals compared to controls, which is why we hypothesized that sleep deprivation may enhance cocaine CPP via the orexin system given the known role of orexin in motivated behavior. CPP is typically done during the light phase and previous experiments have shown that manipulations of the orexin system during the light phase can influence CPP (Sartor and Aston-Jones, 2012). We don’t believe that light phase testing compromised the orexin manipulations because OX1R antagonism was sufficient to prevent cocaine CPP acquisition and reduce the SD-induced increase in CPP acquisition and expression.

6. It is not clear why a one-tailed t-test is used instead of two-tailed for either the CPP results or the weight validation test? Couldn’t animals show aversion (decrease in preference compared to 0 or to control group) and therefore need a two-tailed test?

We used one-tailed T tests since we hypothesized that sleep deprivation would enhance cocaine CPP and OX1R antagonism would reduce feeding thereby reducing weight based on the literature. Specifically, sleep deprivation increases preference for the stimulant methylphenidate in humans (Roehrs et al., 1999) and induces preference to a low dose of amphetamine in mice (Berro et al., 2018). This information has been added to the introduction.

Regarding OX1R antagonism and weight, orexin influences food intake (Sakurai et al., 1998), while SB334867 administration reduces weight under regular chow (Ishii et al., 2005). In response to the comment below (#7) about validation of SB 334867 by weight loss as irrelevant, this experiment has been removed.

We did not test for significance of aversion since the animals were given cocaine immediately prior to entry into the CPP apparatus. Conditioned aversion to cocaine has been shown when cocaine exposure precedes chamber entry by 15 min, while preference is seen when cocaine was given immediately prior to entry or following a 5 min delay (Ettenberg et al., 1999).

7. The “validation of SB334867 dose by weight loss” is somewhat irrelevant given the complicated role of hypocretin peptides and feeding behavior (as opposed to energy homeostasis). This should be removed to the paper, not only as it is confusing and irrelevant to the study, but also in the fact that they did not even elicit the ‘confirmation’ of hypocretin-receptor antagonism resulting in a significant reduction of overall weight. (The authors would have more accurately tested this with feeding behavior following administration of SB334867.)

We agree that feeding is a more direct indicator of SB334867 efficacy and since the OX1R antagonist used in our experiments was not from the same manufacturer from which hydrolysis was noted by Lewin and colleagues (McElhinny et al., 2012) we removed this experiment.

8. Regarding Figure 1: the text states an increase in preference (compared to a hypothetical value of 0) at the 8mg/kg dose in the first CPP test (figure 1), but this does not appear to be indicated on the graph (only a sign difference BETWEEN the groups is shown). Was this increase in preference at 8mg/kg in both groups statistically significant?

The significant difference from 0 for the 8 mg/kg dose was provided in the table. We had tried to avoid duplicating statistical information by providing it only in text or in tables with group differences shown in the figures when present, but this was deemed confusing by both reviewers. We have added symbols for the within group difference from 0 comparison for all doses in the figures and have revised the table to be more complete at the cost of duplicating some information that is also provided within the text.

There was a significant increase in preference between groups at the 8 mg/kg dose, while animals sleep deprived prior to cocaine conditioning trials showed preference (as indicated by a significant difference from 0) at 3, 8, and 15 mg/kg doses. Animals sleep deprived prior to saline conditioning trials showed preference at 15 mg/kg with a failure to achieve preference at 8 mg/kg even though preference values were similar to those of the undisturbed animals in the CPP expression design, likely due to slightly higher within group variability. This variability appears to be driven by a single animal, though the animal is not an outlier. We added a sentence to the results section of Study 1, as follows: ‘Controls (noSD Coc, SD Sal) showed preference at 15 mg/kg but not at 8 mg/kg, possibly due to high variability driven by one animal (not identified as an outlier when using the ROUT method [GraphPad Prism]).’

We also added the following paragraph to the discussion section regarding the unexpected lack of preference to the 8 mg/kg dose in noSD Coc, SD Sal, as follows: ‘Most groups showed preference for the 8 mg/kg dose of cocaine, a dose in which preference is expected (Campbell et al., 2000); however, animals sleep deprived prior to saline conditioning trials (noSD Coc, SD Sal, Study 1) did not reach statistical significance for preference despite the majority of animals showing an increase in time spent on the cocaine-paired side from the pre to post-conditioning tests (8/12). This lack of preference is likely attributable to high variability in preference scores relative to the group average (126.4 +/- 83.6) and is driven by a single animal as can be seen in Figure 1B, though this animal does not qualify as an outlier. As can be seen from the raw data plots with the 8 mg/kg dose across studies, most animals show an increase in relative time in the cocaine-paired side from pre to post-conditioning, though not all animals do so. We cannot explain the source of the individual differences, but these are consistent with individual differences seen with locomotor sensitization to cocaine (Hooks et al., 1991; Allen et al., 2007) and cocaine self-administration (Glick et al., 1994; Griffin et al., 2007).’

Additionally, we modified the results section for Study 1 to better distinguish the general increase in relative time spent on the cocaine-paired side from pre to post-conditioning tests from preference itself and did the same for other studies for consistency. We now use ‘relative time values’ instead of ‘preference’ for pre- and post-conditioning test values for clarity.

8. (continued) Panel B (showing just 8mg/kg group) is superfluous information that can be relayed purely in text. It is also not clear why the 8mg/kg groups are singled from other doses.

We include the relative time of pre and post conditioning test plots (Panel B in figures 1-4) as a way to show raw data and chose the 8 mg/kg dose since it is common across all studies. While unnecessary since the statistical comparison information is provided, we do not think these plots are superfluous since they illustrate variability seen when using this behavioral task. We added the following statement to the methods section ‘For all studies, pre and post-conditioning relative time values are shown (1B, 2B, 3B, 4B) for the 8 mg/kg dose to illustrate variability between animals within each group alongside the general pattern of increased time in the cocaine-paired side.’

9. Regarding Cocaine CPP, Study 2: saline/saline (0mg/kg cocaine) controls should have been included here as the sleep deprivation test was done prior to the final test (which is different than the 0mg/kg testing group from study 1, so not a suitable control).

We agree that the 0 mg/kg control used in Study 1 is not a suitable control for Study 2. The purpose of the saline/saline only control of Study 1 was to determine if sleep deprivation was sufficient to induce preference on its own. An inducement of preference would be seen as a significant increase in time spent on the side in which saline was received following sleep deprivation (i.e. a significant increase from 0). It is unclear how a change in preference would be observed with sleep deprivation immediately prior to the post-conditioning test since both sides are associated with saline, i.e. why would sleep deprivation prior to the post-conditioning test increase time in one saline-paired side over the other? Furthermore, unless most sleep deprived animals spent more time in the same side of the box (for example the side paired with saline on days 1 and 3) an emergence of group preference would likely not be observable. We used an unbiased apparatus such that there was no difference in the average time spent on either side across pre-conditioning tests so there should be no intrinsically preferred side (as could be seen with biased apparatuses that feature one dark and one bright side). Animals that showed an innate preference for one side of the box during the pre-conditioning test were excluded from experiments. When using an unbiased design with an unbiased apparatus, sleep deprivation prior to the post-conditioning test following saline/saline conditioning would be challenging to interpret.

10. Regarding Figure 2: the text again states an increase in preference for the 8mg/kg groups but this is not reflected (if statistically significant) in the graph (Panel C).

There was an asterisk with a line between groups indicating a significant group difference. Based on feedback from reviewer #2, we now use different symbols to distinguish between comparisons and within group difference from 0 comparisons. Group differences are now labeled with a carrot symbol.

10 (continued). And again, Panel B (showing just 8mg/kg group) is superfluous information that can be relayed purely in text. Should be removed from the figure.

As stated above, we believe the raw data plots are useful for showing variability across animals alongside the general pattern of increased time in the cocaine-paired side post-conditioning.

11. It is not clear why two of three doses were chose for the Study 3 and only one dose was chose for study 4 for tests of hypocretin-receptor antagonism. Please explain.

We used OX1R antagonism for 3 and 8 mg/kg doses for Study 3 on the basis that sleep deprivation induced preference to the 3 mg/kg dose and increased preference for the 8 mg/kg dose as found in Study 1 (CPP acquisition design used in Studies 1 and 3). For Study 4, we used OX1R antagonism for the 8 mg/kg dose since sleep deprivation increased preference to this dose but did not include the 3 mg/kg dose since only a non-significant trend towards preference with the 3 mg/kg dose was found in Study 2 (CPP expression design used in Studies 2 and 4). We have added the following statements to the methods sections for Study 3 and 4: ‘The cocaine doses chosen were based on the doses in which there were significant group differences in Study 1 (3, 8 mg/kg).’ and ‘The cocaine dose chosen was based on the dose in which there was a significant group difference in Study 2 (8 mg/kg).’

12. Tables: The organization of the tables is utterly confusing and not acceptable. It would be helpful to match the order of the columns with the order of the bars in related graphs. The headings of the columns do not match the group labels used in legends of figures, and some are errantly labeled all together. All p-values within the table for t-test versus hypothetical preference value of 0 should be report (some are, and some are not). Each unpaired t-test p-value should be reported as table subscript (some are, some are not).

We thank the reviewer for pointing out the incompleteness and confusing nature of the tables. We remade the tables with more complete information resulting in some information repeated in the text and tables, but we believe this change greatly improves readability. We have also added additional statistical information including 95% confidence intervals for all comparisons, difference between means for between group comparisons, and the specific statistical comparisons used. The order of the groups in the tables now matches that of the corresponding figures.

13. The interpretation of data in Figures 3 and 4 of “Orexin antagonism prevented the SD-induced enhancement of both acquisition and expression.” makes no sense without appropriate vehicle controls.

All groups in Study 3 received both the OX1R antagonist and its vehicle control with two of the groups serving as controls (noSD Veh Coc, SD SB Sal and noSD SB Coc, noSD Veh Sal) for the experimental group (SD SB Coc, noSD Veh Sal), though an additional control of noSD Veh Coc, noSD SB Sal could have been added for completeness. We did not include any vehicle control groups in Study 4, which was also raised as a potential weakness by reviewer #2, instead using noSD SB as the control for SD SB. We agree that this is a potential limitation since the Veh could have an effect different than that of no injection (as used in Study 2) and at certain concentrations DMSO (used to dissolve the OX1R antagonist) can influence behavior; however, we diluted DMSO to reduce the concentration below that which behavioral effects are observed (Cavas et al., 2005). We have added a paragraph in the discussion section for potential limitations and include the lack of vehicle only controls as one limitation.

14. Figure 5- Panel B: The most direct test of effects of orexin-receptor antagonism on the enhanced effects of sleep deprivation on cocaine-induced cpp are a comparison between “noSD Coc, SD Sal” (gray) and “noSD Veh Coc, SD SB Sal” (green), and between “SD Coc, noSD Sal” (red) and “SD SB Coc, noSD Veh Sal” (Purple). These statistical analyses (two-way anova) should be carried out and added to Figure 5B (or made into a bar graph with only these four groups). The preference scores of controls, in this case gray and red, may not be significantly different/attenuated by treatment of SB of related treatment groups (green and purple, respectively). This is important information in the interpretation of the data and the role of hypocretin/orexin in sleep deprivation-enhanced cocaine-CPP acquisition. Similar analyses should be completed and interpreted for sleep-deprivation-derived enhancement of established cocaine-CPP.

We did not perform statistical comparisons across studies since there was a considerable time gap between data collected for Study 1 and Study 3 and Study 2 and Study 4 along with some differences in the actual boxes used for CPP conditioning as noted in the methods section. Within each study, control and experimental animals were littermates and data were collected concurrently across groups with multiple sets of control and experimental animals collected for each study; however, there was a considerable time lag between data collection of related studies. We acknowledge that a superior design would include concurrent data collection for related studies (1&3, 2&4) in order to allow direct comparisons instead of the piecemeal design used here. We have included this point in the discussion of potential limitations. We also softened the language within this paragraph by modifying ‘orexin-dependent’ to ‘orexin-influenced’ and ‘prevented’ to ‘reduced’ since these terms are more consistent with observational comparisons.

14 (continued). These results may suggest the need for further dose testing of the SB compound.

The dose of OX1R antagonism used was sufficient to reduce SD-induced enhancement of cocaine CPP. While a higher dose may be able to prevent the acquisition or expression of CPP following SD, as was seen in the absence of SD in Study 3, higher doses would also be expected to reduce locomotor activity (Rodgers et al., 2001) which would be a potential confound.

15. Figure 5- Panel D: What dose are these data derived from? It is also no clear which groups these data are derived from. Statistical analyses should be performed on these data.

Date were derived from Study 2 and 4 in which preference score for the experimental group was normalized to preference score for the control group for each study. We modified the figure legend (Study 2, Study 4) and Y axis label (8 mg/kg) to make the data source clearer. Also, we modified the description of this comparison to provide more details and added a statement to explicitly note that this and other graphs in figure 5 are observational comparisons not statistical comparisons. The end of this paragraph now reads ‘Unexpectedly, OX1R antagonism in undisturbed animals prior to the post-conditioning test [Study 4; noSD SB] led to preference values similar to that of sleep deprived animals in the absence of OX1R antagonism [Study 2; SD], thereby reversing the polarity of the effect of SD as determined by dividing the group average preference score of the sleep deprived group by the group average preference score of the undisturbed group (i.e. [Study 2, SD/noSD]; [Study 4, SD SB/noSD SB]). A score above 1 indicates that SD results in a higher relative preference score compared to the undisturbed condition, while a score below 1 indicates that SD results in a lower relative preference score compared to the undisturbed condition. Statistical comparisons across studies were not performed because of data collection constraints (see discussion section) so these comparisons are observational in nature and should be interpreted with caution.’ This addition also modifies a couple of terms used in the original manuscript that have been revised for accuracy (reversing instead of inverting and polarity instead of direction).

16. Can the authors provide a justification for the use of the 4 hours of sleep deprivation in their model?

We apologize for not including the rationale for the SD duration. Four hours of sleep deprivation was used since this duration reliably induces a homeostatic response as measured by an increase in slow wave activity (SWA, 0.5-4.5 Hz) during slow wave sleep (Bjorness et al., 2018b). Furthermore, this duration does not increase expression of glucocorticoid-related genes as determined by transcriptome analysis of cortical tissue (Bjorness et al., 2020). The Bjorness et al., 2020 citation is a BioRvix preprint; this citation will be updated if this paper is accepted for publication (currently conditionally accepted and under re-review) while the current manuscript is being considered.

17. The Discussion is a very cursory and superficial summary and interpretation of these data. This Discussion needs significant improvement.

We acknowledge that the discussion was quite short, but we tried to avoid speculating much beyond interpretation of the results of the current studies. We have added two new paragraphs based on the concerns raised in this reviewer’s earlier points and an additional paragraph (given below) that puts the findings of the current manuscript in context with related experiments and suggests future experiments that could shed additional light on the ability of SD to enhance the rewarding properties of drugs. ‘The SD-induced enhancement of cocaine CPP is consistent with previous studies in which SD increases preference of methylphenidate in humans (Roehrs et al., 1999) and induces preference to a low dose of amphetamine in rodents (Berro et al., 2018). However, there are several additional studies that would be of interest in further delineating the ability of sleep loss to influence reward. First, thus far all studies have used stimulants so the generalizability of the SD-induced enhancement of preference across drugs is unknown. Additionally, the time course of this enhancement preference is unclear. A long term enhancement of preference would likely be more relevant to the development of addiction than if the SD-induced enhancement is quickly lost. Finally, it is unknown if SD-induced enhancement of stimulant reward is sustained in drug experienced animals since existing studies have included drug naïve rodents or non-dependent humans.’

Reviewer 2

Sleep-mediated regulation of drug reward is increasingly recognized, while the underlying mechanisms are poorly understood. The orexin system represents an important hub that integrates arousal and reward/motivation regulation. This study reports that acute sleep deprivation enhances both the acquisition and expression of cocaine-induced place-preference, and examines the role of orexin receptor activity in regulating this effect. The experiments are thoughtfully designed and carefully conducted. The manuscript is carefully written and mostly clear and logical. There are a few concerns as follows, mostly minor ones.

Results:

1. There is no mentioning of whether changes in CPP may be associated with locomotor activity changes. This may be relevant given orexin’s role in arousal, especially after SD.

We do not believe that the change in CPP is related to locomotor activity changes since it has been previously shown that acute (4h) SD does not influence the magnitude of locomotor sensitization to cocaine (Bjorness and Greene, 2018b). There is no difference in chamber entries during the post-conditioning test with any of the doses for Studies 1, 3, 4 and for 15 mg/kg cocaine for Study 2; we do not have chamber entry information for 3 and 8 mg/kg for Study 2. We also do not have general activity measures outside of chamber entries, itself a relatively gross indicator of activity, for 3 and 8 mg/kg cocaine in Studies 1 and 2 so we cannot directly assess activity in the CPP chambers for the specific conditions in which there is a group difference. For Study 1 with 15 mg/kg cocaine, there was no difference in activity as determined by beam breaks, between animals sleep deprived immediately prior to the first cocaine conditioning trial compared to those that were undisturbed. While we do not believe that locomotor activity increases are associated with the SD-induced enhancement of CPP, we cannot exclude this possibility. We include the following statement in a newly added limitations paragraph: ‘Additionally, since activity measures are not available for conditionings performed with all of the CPP boxes, we cannot exclude a possibility that enhanced preference is associated with an increase in locomotor activity; however, it has been shown that acute SD does not influence the magnitude of locomotor sensitization to cocaine (Bjorness and Greene, 2018b) so an SD-dependent increase in locomotor activity is not expected.’

2. To examine cocaine x sleep interactions, two-way ANOVA is more appropriate than T tests. Cocaine x sleep x SB may need multi-factor ANOVA.

We acknowledge the possibility of falsely finding a significant effect due to the use of multiple t tests instead of using a two-way ANOVA with corrections for multiple comparisons. Many CPP experiments in which a two-way ANOVA is used include phase as a factor such that both pre-conditioning and post-conditioning values are present. This makes it easier to see a significant effect since preference scores should be very low during the pre-conditioning phase. We chose to subtract pre-conditioning values from post-conditioning values to ensure that any increase in preference cannot be attributed to slight preferences in the cocaine-paired chamber prior to drug exposure. We also used an unbiased design such that some animals received cocaine in their initially more preferred compartment which would also serve to limit the possible increase in preference that occurs following cocaine as is apparent in the plots of relative time from pre to post-conditioning (Figures 1B, 2B, 3B, 4B). We have added 95% confidence intervals and difference of means information to the revised tables for each of the statistical comparisons to provide more robust information than p values alone.

There is also no explanation why some used one-tail and others used two-tail T tests (Tables 1-3). ?

We used one-tailed T tests for 3 mg/kg, 8 mg/kg, and 15 mg/kg cocaine with Study 1 and 2 and with weight (now removed based on the suggestion of reviewer #1) since we hypothesized that sleep deprivation would enhance cocaine CPP and orexin-receptor antagonism (OX1R) would reduce feeding thereby reducing weight based on the literature. Conversely, we used two-tailed T tests for comparisons in which there is not direct literature support of the hypothesis (as in the case of the ability of OX1R to influence SD-mediated increases in cocaine CPP). We added information pertaining to the literature support of our hypothesis that SD would enhance cocaine CPP to the introduction, as follows: ‘SD also influences reward in that SD increases preference for the stimulant methylphenidate in humans (Roehrs et al., 1999) and induces preference to a low dose of amphetamine in mice (Berro et al., 2018).’ We also revised the statistical comparisons information in the methods section to clarify statistics used in each Study and provide the rationale for the use of one and two tailed T tests.

Minor concerns:

3. Figure 1: significant differences for within-group and between-group comparisons are both labeled with “*”. This appears confusing and can be changed to use different symbols and be consistent with the other figures (e.g. Figure 2).

We appreciate the reviewer pointing out this confusing oversight and have changed the between-group comparison significant difference symbol to a carrot.

4. Figure 4: There is no control group “Veh noSD” to compare for SB effect alone (on cocaine CPP), in the absence of SD. The authors have cited published studies for this, but it is not clear if the experimental conditions match. If the authors intend to examine an orexin x sleep “interaction", then the design needs to be more complete. Otherwise, the phrasing needs to be revised.

This point was also raised by reviewer #1 and we have added a limitations paragraph to the discussion section in which we discuss the lack of a vehicle-only control group, as follows: ‘First, Study 4 lacked a vehicle control group; a control group of noSD SB was used for the experimental group SD SB in which the ability of OX1R-antagonism to counter SD-induced enhancement of cocaine CPP to an 8 mg/kg dose of cocaine (from Study 2) was tested. DMSO was diluted in order to reduce the concentration below that which behavioral effects are observed (Cavas et al., 2005). However, the effect of vehicle alone on CPP expression was not determined so the possibility that SD-induced enhancement of cocaine CPP was reduced by the vehicle cannot be excluded.’ We also rephrased the introductory statement of Study 4 within the results section from ‘however an interaction with SD’ to ‘however OX1R antagonism following SD’ to more accurately describe the comparison that was conducted.

5. Figure 5: There is no show of statistical significance in this figure.

We do not include statistical significance within the dose-response figure since we did not do statistical comparisons across studies. We added a statement within the results section for this section to explicitly note that the graphs in figure 5 are not statistical comparisons but are instead observational in nature and that they should be interpreted with caution. We also discuss the lack of between study comparisons as a potential limitation within the newly added ‘limitations’ paragraph.

Conclusions (minor):

6. The authors pointed out that “Notably, the relative preference of <1 under orexin antagonism indicates that orexin antagonism in the presence of SD reduces relative preference, while orexin antagonism in the absence of SD increases relative preference.” Therefore, the authors concluded that “Unexpectedly, orexin antagonism..., inverting the direction of the effect of SD.” However, results in Figure 4C shows that in the presence of SB (orexin antagonism), there is no significant difference between SD and non-SD groups, therefore does not support this conclusion. Although Figure 5D shows that SD/noSD ratios are >1 and <1 respectively, there is no statistics to validate this conclusion.

We agree with the lack of statistics and have added a statement that this and other graphs in figure 5 are observational in nature, not based on statistical comparisons such that comparisons should be interpreted with caution. While we did not directly compare outcomes across studies due to data collection constraints, we wanted to visualize that not only did OX1R antagonism reduce the SD-induced enhancement of CPP (as shown in Figure 4C), but also that the group average preference values were non-significantly lower compared to the control group and show this by dividing the preference score of the experimental group by that of the control group. When normalizing the experimental group preference value to that of the control group, polarity is reversed between Study 2 (> 1) and Study 4 (<1). We modified the result description to more explicitly state the transformation being used after which the statement regarding the lack of statistical comparisons is given.

We address the lack of direct comparisons between related studies (Study 1 & 3 and Study 2 & 4 in figure 5) as a potential limitation within the newly added limitations paragraph.

7. The authors concluded that “Orexin antagonism prevented the SD-induced enhancement of both acquisition and expression.” However, in Figure 3C (CPP acquisition), “SD SB Coc” (purple) shows preference to Coc-paired side, whereas “noSD SB Coc” (blue) shows no preference to Coc-paired side, thus it appears that the SD-induced enhancement may be reduced rather than entirely prevented by orexin antagonism.

We agree and revised the results statement to reflect this interpretation as follows: ‘Finally, SB 334867 prevented preference in the absence of SD and blocked SD-induced enhancement of preference to a 8 mg/kg dose of cocaine; however SD animals treated with SB 334867 did show preference for the cocaine-paired side suggesting that SD-dependent enhancement is reduced but not entirely prevented (Figure 3C; Table 3).’ We also changed ‘prevented’ to ‘reduced’ within the section for figure 5 (summary graphs) and discussion section.

Possible typo related to results (minor):

8. “15 mg/kg dose of cocaine (Figure 2C; Table 2), but the sleep deprived group showed a non-significant trend towards preference for the cocaine-paired side.” Likely should be “towards reduced preference for ...”

For the 15mg/kg cocaine dose, the SD group did not achieve preference based on the statistical analysis, but rather approached preference based on the p value (p = 0.1). We heavily revised the tables to provide fuller statistical information, including details that are given elsewhere. We believe that these new tables will make the within and between group comparisons easier to follow.

Citations added to the manuscript:

Allen RM, Everett CV, Nelson AM, Gulley JM, Zahniser NR (2007) Low and high locomotor responsiveness to cocaine predicts intravenous cocaine conditioned place preference in male Sprague-Dawley rats. Pharmacol Biochem Behav 86:37-44.

Bjorness TE, Greene RW (2018b) Sleep deprivation alters the time course but not magnitude of locomotor sensitization to cocaine. Sci Rep 8:17672.

Bjorness TE, Kulkarni A, Rybalchenko V, Suzuki S, Bridges C, Harrington AJ, Cowan CW, Takahashi JS, Konopka G, Greene RW (2020) An essential role for MEF2C in the cortical response to loss of sleep. bioRvix.

Cavas M, Beltran D, Navarro JF (2005) Behavioural effects of dimethyl sulfoxide (DMSO): changes in sleep architecture in rats. Toxicol Lett 157:221-232.

Glick SD, Raucci J, Wang S, Keller RW, Jr., Carlson JN (1994) Neurochemical predisposition to self-administer cocaine in rats: individual differences in dopamine and its metabolites. Brain Res 653:148-154.

Graham DL, Krishnan V, Larson EB, Graham A, Edwards S, Bachtell RK, Simmons D, Gent LM, Berton O, Bolanos CA, DiLeone RJ, Parada LF, Nestler EJ, Self DW (2009) Tropomyosin-related kinase B in the mesolimbic dopamine system: region-specific effects on cocaine reward. Biol Psychiatry 65:696-701.

Hooks MS, Jones GH, Smith AD, Neill DB, Justice JB, Jr. (1991) Individual differences in locomotor activity and sensitization. Pharmacol Biochem Behav 38:467-470.

McClung CA, Sidiropoulou K, Vitaterna M, Takahashi JS, White FJ, Cooper DC, Nestler EJ (2005) Regulation of dopaminergic transmission and cocaine reward by the Clock gene. Proc Natl Acad Sci U S A 102:9377-9381.

Roehrs T, Papineau K, Rosenthal L, Roth T (1999) Sleepiness and the reinforcing and subjective effects of methylphenidate. Exp Clin Psychopharmacol 7:145-150.

Citations used in the response but not added to the manuscript:

Bardo MT, Rowlett JK, Harris MJ (1995) Conditioned place preference using opiate and stimulant drugs: a meta-analysis. Neurosci Biobehav Rev 19:39-51.

Ettenberg A, Raven MA, Danluck DA, Necessary BD (1999) Evidence for opponent-process actions of intravenous cocaine. Pharmacol Biochem Behav 64:507-512.

Griffin WC, 3rd, Randall PK, Middaugh LD (2007) Intravenous cocaine self-administration: individual differences in male and female C57BL/6J mice. Pharmacol Biochem Behav 87:267-279.

Liu X, Chen Y, Tong J, Reynolds AM, Proudfoot SC, Qi J, Penzes P, Lu Y, Liu QS (2016) Epac Signaling Is Required for Cocaine-Induced Change in AMPA Receptor Subunit Composition in the Ventral Tegmental Area. J Neurosci 36:4802-4815.

Rodgers RJ, Halford JC, Nunes de Souza RL, Canto de Souza AL, Piper DC, Arch JR, Upton N, Porter RA, Johns A, Blundell JE (2001) SB-334867, a selective orexin-1 receptor antagonist, enhances behavioural satiety and blocks the hyperphagic effect of orexin-A in rats. Eur J Neurosci 13:1444-1452.

Schindler AG, Messinger DI, Smith JS, Shankar H, Gustin RM, Schattauer SS, Lemos JC, Chavkin NW, Hagan CE, Neumaier JF, Chavkin C (2012) Stress produces aversion and potentiates cocaine reward by releasing endogenous dynorphins in the ventral striatum to locally stimulate serotonin reuptake. J Neurosci 32:17582-17596.
